# Long-Term Durability of Tunneled Hemodialysis Catheters: Outcomes from a Single Institution 22-Year Experience

**DOI:** 10.1007/s00270-024-03941-4

**Published:** 2025-02-04

**Authors:** Austin Zhang, Timothy WI Clark, Scott O. Trerotola

**Affiliations:** 1 Perelman School of Medicine at the University of Pennsylvania, Philadelphia, PA USA; 2 Department of Radiology, Division of Interventional Radiology, Perelman School of Medicine at the University of Pennsylvania, Philadelphia, PA USA

**Keywords:** Tunneled hemodialysis catheter, Durability, Repair

## Abstract

**Purpose:**

To describe long-term physical durability of tunneled hemodialysis catheters, highlighted in the 2019 Kidney Disease Outcomes Quality Initiative (KDOQI) guidelines as a specific area for future research.

**Materials and Methods:**

Tunneled hemodialysis catheters with known outcomes and dwell times > 1 year were entered into this retrospective study. Data includes demographics, complications, catheter type, dwell time, reason for removal, access site, and placement via exchange or de novo. Catheter durability < 1 year dwell was analyzed as a secondary aim.

**Results:**

272 catheters in 229 patients were included. Dwell times ranged from 366 to 3,802 days (median 504), totaling 162,439 catheter days. 17 (6%) catheters > 1 year dwell had broken external components. For these, dwell times until breaking ranged from 377 to 1,436 days (median 489), totaling 10,434 catheter days. 5 had a broken hub, 11 had a broken clamp, and 1 had broken hub and clamp. 12 were Ash Split Cath (n = 240) and 5 were Arrow-Clark VectorFlow (n = 32). In the durability < 1 year sub-analysis, 6,515 catheters with dwell times < 1 year in 3,693 patients were included, totaling 425,018 catheter days. 48 were damaged, with 24 broken hubs, 17 broken clamps, and 7 holes. Median time to breakage was 110 days. 38 were Ash Split Cath (n = 5,636) and 10 Arrow-Clark VectorFlow (n = 812). In both analyses, breakage was limited to hubs, clamps, and extensions.

**Conclusions:**

Tunneled hemodialysis catheters are exceptionally durable, rarely requiring removal for hub-related issues after one year. Breakdown was not observed as a long-term durability issue. Further, broken external components can be replaced using external repair kits.

**Level of Evidence:**

Level 2b, Retrospective Study.

**Graphical Abstract:**

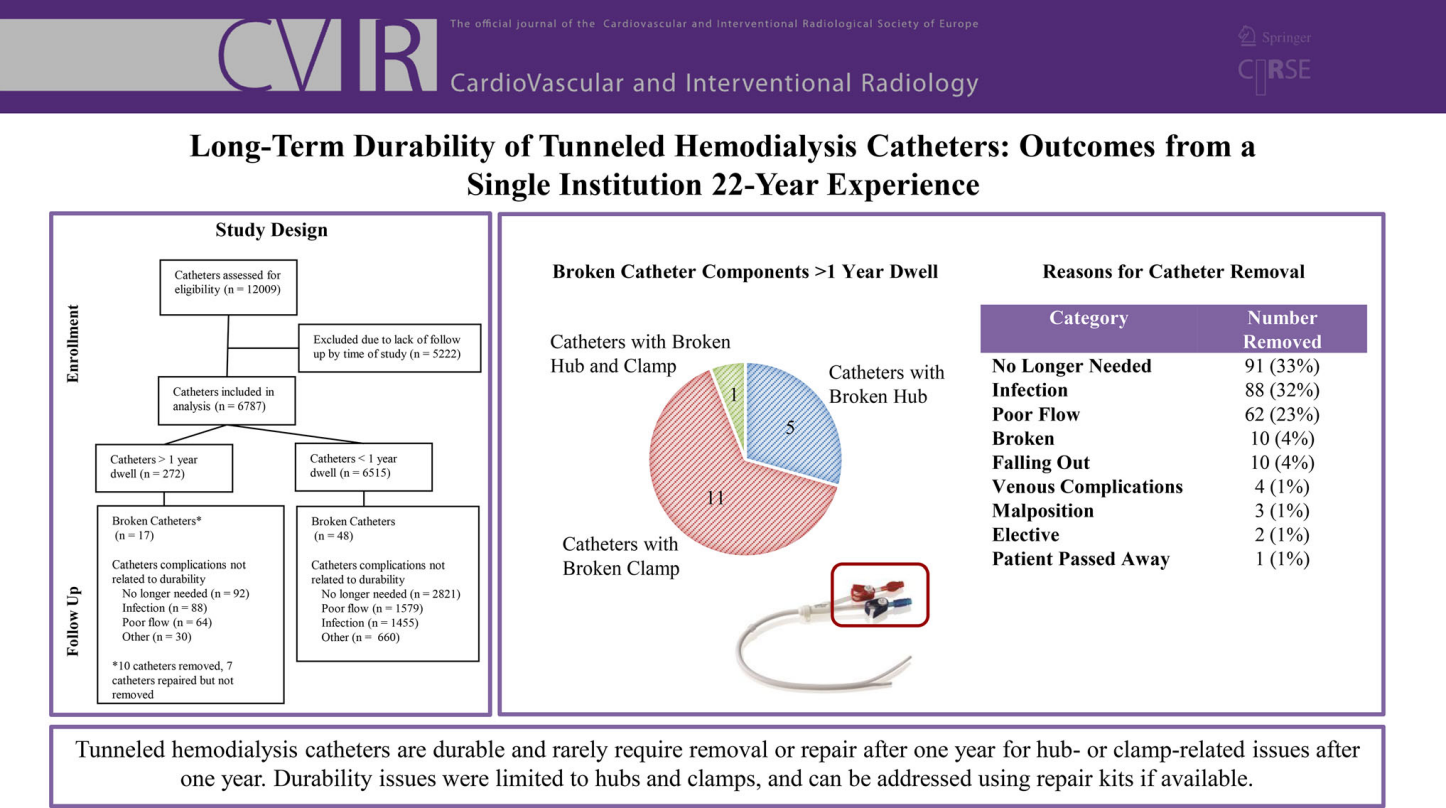

## Introduction

Three forms of vascular access are most widely used for hemodialysis: arteriovenous fistulas, arteriovenous grafts, and tunneled dialysis catheters (TDC). Fistulas and grafts are preferred over TDCs because they are associated with fewer complications such as infection, thrombosis, central venous stenosis, and breakage [1, 2]. However, many patients rely on TDCs for their long-term care because of difficulties establishing permanent access, limited access options, or patient preference [3, 4]. In the US, 81% of patients with end-stage renal disease (ESRD) initiate hemodialysis with a catheter. 36% of patients with ESRD continue to require a TDC 6 months after hemodialysis initiation, and 17% continue to require a TDC 12 months after hemodialysis initiation [5].

Multiple approaches have been taken to improve catheter longevity: heparin and antibiotic coatings have been developed to prevent thrombosis and infection, and lumens have been designed to preserve patency [6, 7]. However, the long-term physical durability of the catheter material itself has been poorly characterized. Indeed, the National Kidney Foundation’s 2019 KDOQI Vascular Access Guidelines recommends long-term catheter durability as a specific area for future research [8]. To better understand long-term durability of TDCs, a retrospective study of long-term TDC use at a single institution was conducted.

## Materials and Methods

### Patient Population

**Fig. 1:**
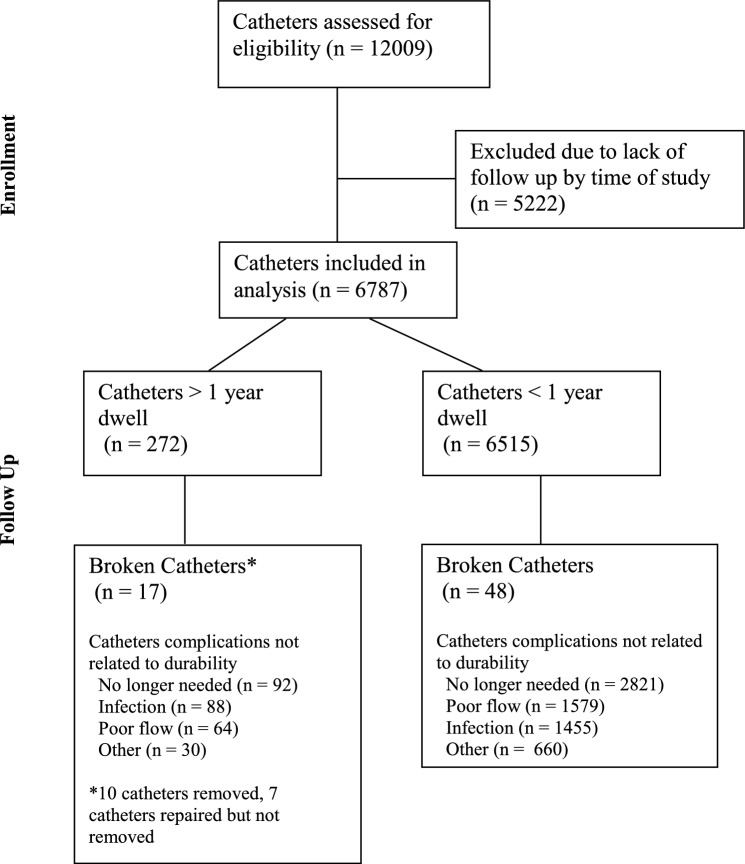
Catheter Groups Analyzed

Institutional review board approval was obtained for this retrospective study, performed in full compliance with the Health Insurance Portability and Accountability Act. Using a quality assurance database, tunneled hemodialysis catheters placed by the Division of Interventional Radiology at two different hospitals in the same health system were catalogued over a 22-year period from 2001 to 2023. Catheters with dwell times of greater than 1 year were identified and entered into this study to examine long-term durability. Catheters with dwell times of less than 1 year were additionally identified and entered into this study for a sub-analysis to ensure mechanical failures occurring in the first year were captured. Only catheters with known follow-up were included. For each catheter, patient demographics, complications, and catheter related data were recorded. Catheter related data include catheter type, dwell time, reason for removal, access site, and whether catheters were placed de novo or via exchange over guidewires. A diagram detailing inclusion and exclusion of catheters is provided in Fig. 1.

### Catheter Types and Placement

Table 1 shows catheter types and lengths. Two different catheter types were used, AshSplit (Medcomp, Harleysville, PA) and Arrow-Clark VectorFlow (Teleflex, Morrisville, NC). The 14F AshSplit is made of polyurethane, is available in antegrade design, has dual lumens, has a repair kit available, and has mean flow rates of 299–413 mL/min [9]. The 15F Arrow-Clark VectorFlow is made of polyurethane, is available in antegrade or retrograde design, has dual lumens, has a repair kit available for the retrograde version of the catheter, and has mean flow rates of 333–392 mL/min [10]. Choice of catheter was at the discretion of the inserting attending interventional radiologist. Catheters were placed by attending interventional radiologists with 1 to 30 years of experience in TDC placement, or by interventional radiology trainees under the direct supervision of an attending interventional radiologist. All catheters were placed under sterile conditions with real-time ultrasound guidance for venous puncture and fluoroscopic guidance for catheter placement. Broken catheter components were replaced if possible by using commercially available repair kits (MedComp) or were exchanged if repair kits were not available (there is no repair kit for the VectorFlow used in this study). Catheter exchange was performed over 1 (usually) or 2 hydrophilic guidewires under fluoroscopic guidance. Antibiotic prophylaxis was not given for de novo placement but was given for exchange (usually cefazolin 1 g IV).

**Table 1:** Catheter Types and Lengths

Catheter Type	Number of Catheters	Total (%)
Ash 24 cm 28 cm 32 cm 36 cm 55 cm	21 163 53 1 2	240 (88%)
VectorFlow 19 cm 23 cm 27 cm	7 19 6	32 (12%)

### Reasons for Removal or Exchange

Complications resulting in catheter removal or exchange were recorded at time of presentation. The reasons for removal were categorized as broken, poor flow, infection, no longer needed, falling out, mispositioned tip, and venous complications such as arm swelling. Individual reasons for removal and their corresponding category groupings are provided in Table 2. One patient in the study passed away, marking the end of that catheter’s dwell-time which is also represented in Table 2.

**Table 2:** Reasons for Catheter Removal

Category	Number Removed	Total (%)
No Longer Needed Mature Access Transplant Transfer to Peritoneal Dialysis (CAPD) Recovered Withdrawing Dialysis/Hospice	68 12 5 6 1	92 (34%)
Infection Bacteremia Sepsis Infection Tunnel Infection Fungemia	67 12 5 3 1	88 (32%)
Poor Flow	64	64 (24%)
Broken	10	10 (4%)
Falling Out	10	10 (4%)
Venous Complications Arm Swelling Venous Stenosis Thrombosis	2 1 1	4 (1%)
Mispositioned Tip	3	3 (1%)
Patient Passed Away	1	1 (1%)

### Outcome Measures

Catheters were followed from date of placement until date of removal or exchange. Complications resulting in catheter removal or exchange were examined. Catheter repairs that occurred more than 1 year after a catheter was placed were additionally identified.

Rates of complications were calculated per 1,000 catheter days. The term “catheter days” refers to the number of days that catheters are indwelling across a sample of patients.

## Results

272 tunneled hemodialysis catheters in 229 patients with dwell times of greater than 1 year were identified. Of these, 240 were Ash Split and 32 were Arrow-Clark VectorFlow. For these catheters, the shortest dwell time was 366 days, and the longest was 3,802 days (median 504 days, mean 598 days). The data cover a total of 162,439 catheter days. Overall, the rate of complications requiring removal or exchange was 1.67 per 1,000 catheter days.

Most catheters were removed or exchanged because they were no longer needed, were infected, or had poor flow. 92 catheters (33.8%) were no longer needed because of maturation of arteriovenous access, 88 (32.4%) were removed due to infection, and 64 (23.5%) had poor flow. A complete list of complications can be found in Table 2.

Catheters were placed in the following access veins: right internal jugular (RIJ), right external jugular (REJ), left internal jugular (LIJ), left external jugular (LEJ), collateral veins, right femoral, and left femoral. Most catheters were placed in the RIJ (197, 72.4%) and LIJ (56, 20.6%). A complete list of catheter placements by access vein can be found in Table 3.

**Table 3:** Catheter Placement by Access Vein

Access Vein	Number of Catheters
Right Internal Jugular Right External Jugular Left Internal Jugular Left External Jugular Collateral Right Femoral Left Femoral	197 12 56 3 2 1 1

Of the 272 catheters, 157 were placed de novo (57.7%) and 113 (41.5%) were exchanged over guidewires. 2 (0.7%) catheters were placed after tract recanalization.

### Catheters with Broken External Components:

A total of 17 (6.3%) catheters had external component breakage after more than 1 year of dwell time. 10 of these catheters were removed, and 7 were repaired. All the repaired catheters were Ash Split Cath. Dwell times until repair or removal ranged from 377 to 1,436 days (median 489 days, mean 613 days). 5 had a broken hub, 11 had a broken clamp, and 1 had both a broken hub and broken clamp. 12 were Ash Split Cath and 5 were Arrow-Clark VectorFlow. The rate of broken components was 5.0% for Ash Split Cath and 15.6% for Arrow-Clark VectorFlow. With regards to placement location, 10 were placed in the RIJ, 5 in the LIJ, and 2 in collateral veins. 8 were placed de novo, and 9 were placed via exchange over guidewire. Of the catheters placed via exchange, 7 were Ash Split Cath and 2 were Arrow-Clark VectorFlow.

### Sub-analysis of Catheters Below 1 Year Dwell Time with Broken External Components:

6,515 catheters in 3,693 patients with dwell times of less than 1 year were identified, covering a total of 425,018 catheter days. Ash Split Cath accounted for 5,636 of the catheters, and Arrow-Clark VectorFlow accounted for 812 of the catheters. A total of 48 broken catheters were identified, 38 being Ash Split Cath and 10 being Arrow-Clark VectorFlow. 24 had a broken hub, 17 had a broken clamp, and 7 had a hole. The rate of broken external components < 1 year was 0.7% for Ash Split and 1.2% for Arrow-Clark VectorFlow. Median time to breakage was 110 days, and average time to breakage was 125 days.

## Discussion

Many patients with chronic kidney disease rely on tunneled hemodialysis catheters for their long-term care when more ideal forms of access such as fistulas or grafts are not feasible. In this study, the long-term durability of tunneled hemodialysis catheters at a single institution was examined.

Out of 272 total catheters with greater than 1 year dwell, 17 (6.3%) catheters had breakage. This is a small portion of all catheters in the study, demonstrating that overall long-term durability of tunneled hemodialysis catheters is very good. Notably, it was found that only breakage of catheter hubs and clamps accounted for mechanical failure of TDCs. Damage to the polyurethane tubing portion of the catheters was not observed. From a safety standpoint, this is very reassuring since failure of the catheter tubing might be associated with bleeding and/or air emboli, whereas hub and clamp issues are far less likely to have such problems. Indeed, since hub and clamp are redundant when it comes to aerostasis and hemostasis, only the combination of both hub and clamp breakage would expose a patient to the risk of air emboli or bleeding. These findings also underscore that hubs and clamps are especially vulnerable sites of mechanical breakage, which might be an opportunity for future improvements to TDCs. As many manufacturers of vascular catheters source external components from third-party vendors known as OEMs (original equipment manufacturers), these findings also illustrate the need for robust quality assurance systems within the manufacturing and assembly process to ensure the most robust components are utilized for vascular access devices [11].

In the sub-analysis of catheter durability under 1 year of dwell, 48 (0.7%) catheters had broken external components out of 6,515 total catheters. Compared to the greater than 1 year dwell catheter group, this sub-analysis group had an eightfold lower rate of component breakage. The rate of component breakage encountered in our sub-analysis of TDCs below 1 year dwell time is comparable to that found in prior studies of TDC outcomes. In Alomari and Falk’s study that tracked outcomes of 344 TDCs with a median dwell time of 66 days, there were 5 (1.5%) cracked catheters [12]. In a similar study of TDC outcomes by Trerotola et al. of 250 catheters with median dwell time of 56 days, there were 3 (1.2%) broken catheters [13].

Fortunately, hubs and clamps can be repaired on an outpatient basis, at the bedside, or even in the dialysis unit if there is a repair kit available, mitigating the need to remove and replace an entire catheter. A repair kit currently exists for the Ash Split Cath. There is also a recently released repair kit for the Arrow-Clark VectorFlow, but it only covers the version of the catheter with a retrograde tunnel design, which is not used at this institution [14]. However, it is possible that the retrograde repair kit might be able to be used to repair antegrade placed TDCs of this type. The lack of a repair kit might be an important consideration when placing TDCs, especially for TDCs with anticipated long-term use.

Repair is a safe and effective way to extend catheter life without increasing infection risk. Although literature regarding hemodialysis central venous catheter durability is sparse, there are a few studies describing catheter repair outcomes in the parenteral nutrition realm. Wouters et al. conducted a retrospective analysis of 527 silicone Hickman catheters (BD Bard, Murray Hill, NJ) for home parenteral nutrition and identified 58 (11.0%) repairs across 41 catheters [15]. Repair extended catheter survival by 510 days (median time to first repair was 452 days), effectively doubling catheter survival. They found that damage most frequently occurred in the distal catheter segment to the adapter or clamping sleeve, often caused by screw thread failure or friction between the rigid adapter and flexible clamping sleeve. These findings are similar to the catheter vulnerabilities discovered in the present study. However, damage to the catheter tubing itself was additionally observed in the Wouters et al. study but was not observed in the present study. This may reflect a difference in catheter material, with the catheters in the study of Wouters et al. being silicone and those in the present study being thermoplastic polyurethane, a more durable material. Of note, although there was a shift in TDC material from silicone to polyurethane through the 1980s and 1990s, since then there have been no further changes in catheter material as polyurethane has become preferred [16].

## Limitations

Our study is limited by its retrospective design. A large number of catheters was lost to follow-up, reflecting an urban care environment and multiple practitioners providing similar services in the same geographic area. Additionally, this study reports outcomes from a single institution and thus results may not generalize to other medical centers. However, the results are consistent with similar shorter-term studies in the literature.

## Conclusion

Tunneled hemodialysis catheters are durable and rarely requiring removal or repair for hub- or clamp-related issues after one year. Catheter hubs and clamps are mechanically vulnerable and were the only sites of breakage found in catheters with greater than 1 year dwell time. Catheter breakage was not observed as a long-term durability issue, and the broken external components identified in this study can frequently be fixed using repair kits. Further, while one-third of the catheters in our study were removed because they were no longer needed, two-thirds of the catheters were removed of complications. The high rate of complications resulting in removal affirms that long-term catheters should be avoided if possible.
